# An unusual case of plasmablastic lymphoma presenting as dermatomyositis

**DOI:** 10.1002/ccr3.2787

**Published:** 2020-03-10

**Authors:** Ashlyn Rui Yin Chee, Teng Fong Ng, Stephanie Jin Ping Lam, Matthew Wright, Michael F. Leahy

**Affiliations:** ^1^ Department of Haematology Royal Perth Hospital Perth WA Australia; ^2^ Department of Haematology Fiona Stanley Hospital Murdoch WA Australia; ^3^ Present address: Department of Haematology Sir Charles Gairdner Hospital Nedlands WA Australia

**Keywords:** autologous stem cell transplant, bortezomib, dermatomyositis, plasmablastic lymphoma

## Abstract

We report a rare case of aggressive plasmablastic lymphoma with an initial presentation of dermatomyositis. The challenges associated with the diagnosis and treatment approach are also highlighted in this case report.

## Graphical abstract

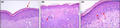

## 1 BACKGROUND

Plasmablastic lymphoma (PL) is a rare and aggressive disease with overlapping features of multiple myeloma and high‐grade B‐cell lymphomas. It is commonly associated with immunodeficiency disorders including the human immunodeficiency virus (HIV) infection.^1^ The disease has a male predominance, and the median age at presentation is 42 years in HIV‐infected patients and 55 years in non–HIV‐infected patients.^1^ The overall prognosis of PL is generally guarded with a reported median overall survival of 6‐19 months, without clear differences between HIV‐positive and HIV‐negative patients.^2^ Given its rarity, there is also currently no accepted treatment approach.^1^ Dermatomyositis is an inflammatory myopathy, which usually manifests as a subacute proximal myopathy, systemic inflammatory process, and a characteristic skin rash, with a 32% risk of cancer diagnosed within 3‐5 years after the onset of the disorder.^3^ Commonly associated cancers include carcinomas of the breast, colon, ovaries, nasopharynx, melanoma, and non‐Hodgkin's lymphoma.^3^ Histologically, it is characterized by atrophic fibers at the peripheral muscle fascicles due to microinfarcts and hypoperfusion, which is mediated by the early activation of the complement C5b‐9 membrane attack complex by antibodies.^3^ Steroids are typically the mainstay of treatment.

## 2 CASE PRESENTATION

A 52‐year‐old male presented with a 3‐week history of rash, which started in his right thigh and later evolved into an acute widespread eruption. This was associated with severe myalgias, arthralgias, lethargy, and chills. He also developed painful mouth ulcers resulting in reduced oral intake and a 6 kg weight loss over 3 weeks. His background medical history includes hypertension and type 2 diabetes, managed with ramipril and metformin, respectively. There was no history of autoimmune disorders, and he was a nonsmoker and nondrinker.

On examination, his rash was an erythematous papulosquamous eruption with desquamation, which was violaceous over his peripheries. Some areas appeared eczematous with serous crusting. Holster's sign was positive and a subtle shawl sign was present, but Gottron's papules and a heliotrope rash were not present.^4^ He had mild proximal myopathy with reduced power (grade 4/5) of his bilateral upper and lower limb proximal muscles. Apart from a tender left wrist, there were no other tender joints or evidence of synovitis. Cardiovascular, respiratory, and abdominal examination were unremarkable.

## 3 INVESTIGATIONS

**Figure 1:**
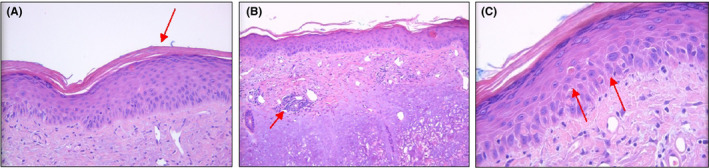
(a) Mild keratosis. (b) Mild sperficial perivascular lymphocytic inflammation. (c) Cytoid bodies (necrotic keratinocytes) and basal vacuolar change. [As indicated by the red arrows]

His initial full blood picture revealed a normal hemoglobin of 132 g/L, neutrophilia with white cell count of 15.7 × 10^9^/L, and a thrombocytosis with a platelet count of 465 × 10^9^/L. His inflammatory markers were elevated with a C‐reactive protein (CRP) of 220 mg/L and erythrocyte sedimentation rate (ESR) of 90 mm/h ANA was slightly elevated at 15 IU/mL with a speckled pattern. His serum creatinine kinase was extremely high at 10 300 U/L (ref interval 45‐250 U/L). Serum lactate dehydrogenase (LDH) was elevated at 1090 U/L (ref interval 120‐250 Us/L). Serum electrophoresis did not detect any paraprotein or elevated serum free light chains, and calcium and creatinine levels were within the normal range. HIV, hepatitis B, and C serologies were negative. A skin biopsy demonstrated an interface dermatitis, consistent with connective tissue disease/dermatomyositis (Figure 1)*.* Magnetic resonance imaging (MRI) of his pelvic muscles was consistent with features of myositis, showing high‐intensity signal and patchy muscle edema of the hip adductors, iliopsoas, and gluteus minimus muscles in a bilateral symmetrical fashion. Lytic lesions were not identified on skeletal imaging. A myositis panel of associated autoantibodies returned negative, and EMG and muscle biopsy were not performed.

**Figure 2:**
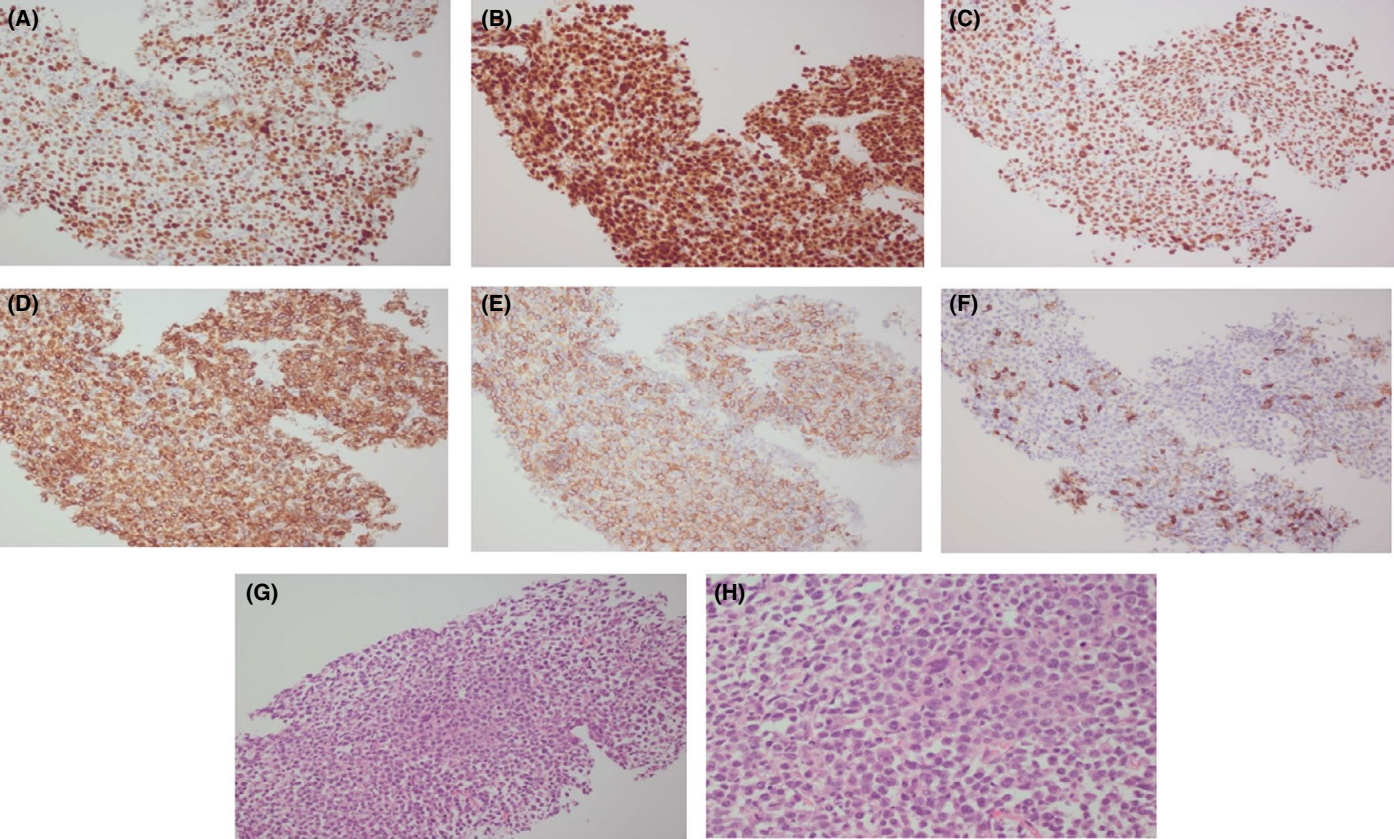
(a) Positive p53. (b) Positive MUM1. (c) Positive ki67. (d) Positive CD138. (f) Negative CD20. (g) H&E (×200). (h) H&E (×400)

**Figure 3:**
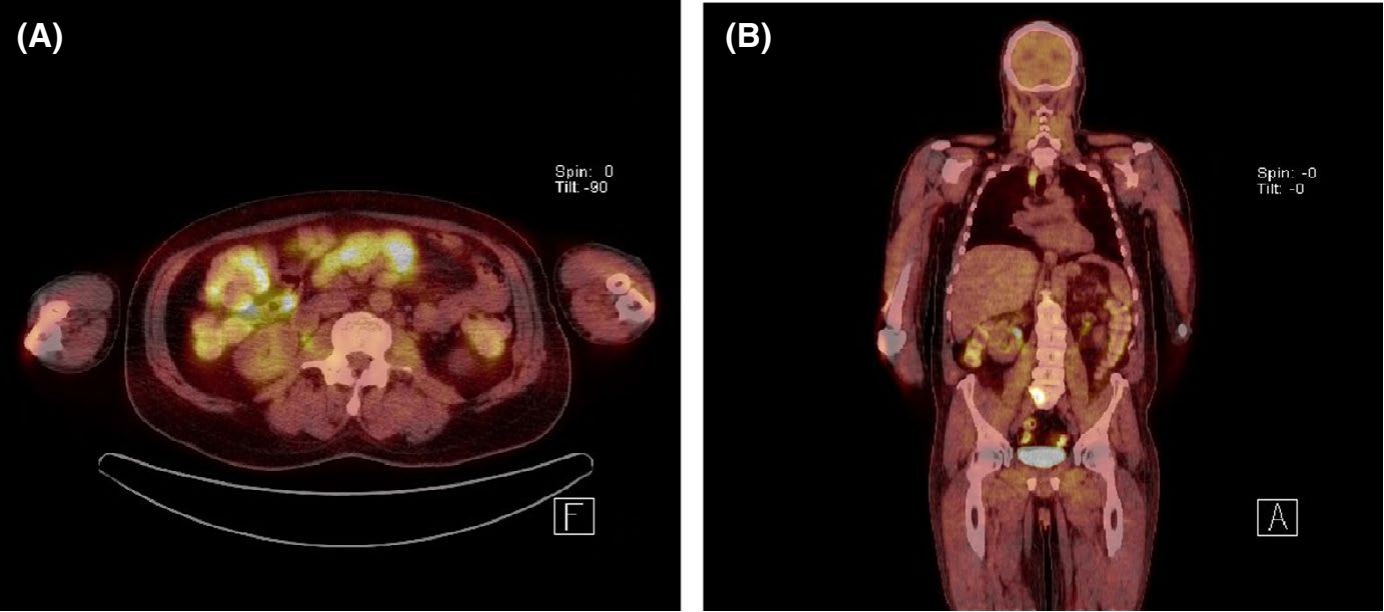
(a) The most active nodal disease is in the central small bowel mesentery (SUVmax 8.7). (b) Nodal involvement below and above the diaphragm and L3 bone involvement

Given the association between dermatomyositis and malignancy, a whole‐body computed tomography (CT) scan was performed, revealing multiple sites of lymphadenopathy. A percutaneous omental lymph node biopsy showed sheets of eccentric blastoid lymphocytes. Immunophenotype was positive for MUM1, CD79a, and CD138, with Ki67 proliferation index of 100%, and negative for leukocyte common antigen CD45, and B‐cell markers CD19 and CD20 (Figure 2). Immunohistochemistry was equivocal (30%) for C‐MYC expression and negative for the Epstein‐Barr encoding region (EBER), HHV8, cyclin D1, CD56, IgA, and IgM. Fluorescence in situ hybridization (FISH) analysis demonstrated a loss of TP53 and no MYC or IgM disruption. A monoclonal B‐cell population was not detected on flow cytometry. Cytogenetics and molecular studies were not performed on the lymph node biopsy. Positron emission tomography (PET) scan was consistent with stage IV disease, with significant small bowel and extranodal osseous involvement (Figure 3). Bone marrow and CSF staging were negative. Given the absence of myeloma‐defining signs, the presence of lymphadenopathy, and the morphological appearance of plasmablasts, a diagnosis of plasmablastic lymphoma was made despite the absence of EBER and negative HIV status.

## 4 TREATMENT

Treatment was started with the dose‐adjusted EPOCH chemotherapy regimen, which consisted of etoposide, prednisolone, vincristine, cyclophosphamide, and doxorubicin.^5^ This was given in combination with bortezomib (V) at 1.3 g/m^2^ given on days 3 and 6 of each cycle. Six cycles of V‐DA‐EPOCH every 21 days were administered, with dose escalation up to the 2nd scale. The patient was also continued on a weaning dose of prednisolone for the treatment of dermatomyositis. As his CNS International Prognostic Index score for lymphoma^6^ was an intermediate risk, prophylactic intrathecal chemotherapy was not administered. His rash and myalgia improved with steroid use and chemotherapy, and LDH and CK levels normalized with treatment. He developed grade 2 peripheral neuropathy affecting his lower limbs by Common Terminology Criteria for Adverse Events (CTCAE) and expected myelosuppression due to the cytotoxic agents.

## 5 OUTCOME AND FOLLOW‐UP

The patient achieved complete metabolic remission, evident on his interim and postinduction chemotherapy PET scans. The patient went on to have consolidation treatment with BEAM (carmustine, etoposide, cytarabine, and melphalan) conditioning protocol and autologous stem cell transplantation. Repeat PET scan at day 100 after his autologous stem cell transplantation showed continuing complete remission of the disease. Unfortunately, he developed florid relapse with brainstem and bone marrow involvement 16 months after his autologous stem cell transplant and was eventually palliated.

## 6 DISCUSSION

Dermatomyositis as a presentation of PL has not been reported previously. More commonly, the site of disease involvement in both HIV‐positive and HIV‐negative patients diagnosed with PL is the oral cavity or jaw, followed by the gastrointestinal tract, lymph nodes, and skin.^1^, ^7^ Most cases present with advanced stage III or IV disease according to the Ann Arbor Staging.^7^ Analysis from the Lymphoma Study Association (LYSA), the largest cohort reported to date, has reported 7 of 58 non–HIV‐related PL cases to be associated with autoimmune conditions.^8^ These included sarcoidosis, inclusion body myositis, severe cutaneous psoriasis, graves’ disease, giant cell arteritis, and two cases of rheumatoid arthritis.^8^ Overall, there was a high incidence of immunosuppression among those with PL, with HIV‐positive patients being the most prevalent and only 5% not showing evidence of immune modulation.^8^ On the other hand, it has been well established that dermatomyositis is strongly associated with a variety of malignancies, which occur primarily as a paraneoplastic syndrome.^9^ The pathophysiologic mechanism linking systemic autoimmunity and malignancies is not well defined, but it has been postulated that antibodies produced against tumor antigens cross‐react with antigens temporarily expressed during normal muscle regeneration and cause muscle inflammation.^9^, ^10^

Immunophenotypically, PL expresses plasma cell markers such as CD138, CD38, and IRF4/MUM1 and loses conventional B‐cell markers such as CD19 or CD20.^1^, ^7^, ^11^ The cell of origin is believed to be from a plasmablast, which is an activated B cell in the process of becoming a plasma cell.^1^ Although the pathogenesis of PL is not entirely known, the MYC gene rearrangement is commonly observed^1^, ^7^, ^11^ and identified in 50% of patients.^11^ In addition, in situ hybridization for EBER is also positive in up to 75% of cases.^1^, ^11^ The diagnosis of this disease is difficult not only due to its unique immunophenotype but also due to several overlapping morphological and phenotypical features with plasmacytoma, plasmablastic myeloma, or diffuse large B‐cell lymphoma (DLBCL). Although there may be several distinguishing features that aid in differentiating these lymphoid tumors, a specific histopathological diagnosis is not always straightforward.^12^, ^13^ Importantly, clinical correlation such as the presence of lymphadenopathy may favor the diagnosis of plasmablastic lymphoma over a plasma cell neoplasm and the presence of end‐organ damage; that is, CRAB criteria or plasma paraprotein may be more suggestive of multiple myeloma.^12^

In the WHO classification, PL has been classified as an aggressive subtype of DLBCL, but the National Comprehensive Cancer Network (NCCN) has reported that standard treatment with CHOP chemotherapy, consisting of cyclophosphamide, doxorubicin, vincristine, and prednisolone, is inadequate and associated with dismal outcomes.^11^, ^14^ Intensive regimens such as HyperCVAD, CODOX‐M/IVAC, and DA‐EPOCH that have higher doses and additional cytotoxic agents such as methotrexate, etoposide, or cytarabine have also not improved survival rates.^11^, ^14^ Majority of patients die within the first year after initial presentation.^11^, ^14^

Recently, studies have shown that bortezomib, a proteasome inhibitor used in the treatment of multiple myeloma, has a therapeutic role in PL on the basis of the plasmacytic differentiation of the plasmablastic cells.^1^, ^11^ As the PL cells are characterized by the expression of plasma cell transcription factors such as XBP1 and BLIMP1, bortezomib may work by targeting these factors, which may have an effect on antibody production and unfolded protein response.^14^ Bortezomib, in combination with DA‐EPOCH, has been reported to induce a complete remission rate of >90% (n = 16) and a 65% 5‐year overall survival in the frontline setting in a retrospective case series published in 2018.^15^ A systematic review of bortezomib in plasmablastic lymphoma also reported that the overall response rate (ORR) to bortezomib‐based regimens was 100%, with complete remission (CR) in 89% of patients and a 2‐year overall survival rate of 55%.^16^

High‐dose chemotherapy as myeloablative conditioning therapy supported by an autologous stem cell transplant has a role as a consolidative treatment^1^ and should be considered in the frontline setting in those who have a MYC gene rearrangement, TP53 gene deletion, age‐adjusted international prognostic index greater or equal to 2, and a partial response or refractory disease.^17^ Based on a review of 9 case reports of HIV‐negative patients with PL, BEAM was the most commonly used conditioning regimen prior to an autologous stem cell transplant.^17^ There is currently no report in the literature with regard to allogenic transplants in HIV‐negative patients with PL.^17^

Plasmablastic lymphoma is a rare and aggressive hematological malignancy that affects both immunocompetent and immunocompromised patients, which often presents at a late stage and associated with poor outcomes. Not only is PL a difficult disease to diagnose, our patient also did not have the typical risk factors that are associated with this condition. This case illustrates the unique presentation of an uncommon disease and the complexities surrounding therapeutic management.

## CONFLICT OF INTEREST

The authors state that there are no conflicts of interest to disclose.

## AUTHOR CONTRIBUTION

AC: wrote and edited the manuscript. TN: contributed to the editing of the manuscript and was involved in the patient's care. ML: was the lead physician and contributed to the editing of the manuscript. MW: was the transplant physician involved in the patient's care. SL: was involved in the patient's care.
